# Neurobiological Signatures of Alcohol Dependence Revealed by Protein Profiling

**DOI:** 10.1371/journal.pone.0082656

**Published:** 2013-12-16

**Authors:** Giorgio Gorini, Amanda J. Roberts, R. Dayne Mayfield

**Affiliations:** 1 Waggoner Center for Alcohol and Addiction Research, The University of Texas at Austin, Austin, Texas, United States of America; 2 Molecular & Cellular Neuroscience Department, The Scripps Research Institute, La Jolla, California, United States of America; CNRS UMR7275, France

## Abstract

Alcohol abuse causes dramatic neuroadaptations in the brain, which contribute to tolerance, dependence, and behavioral modifications. Previous proteomic studies in human alcoholics and animal models have identified candidate alcoholism-related proteins. However, recent evidences suggest that alcohol dependence is caused by changes in co-regulation that are invisible to single protein-based analysis. Here, we analyze global proteomics data to integrate differential expression, co-expression networks, and gene annotations to unveil key neurobiological rearrangements associated with the transition to alcohol dependence modeled by a Chronic Intermittent Ethanol (CIE), two-bottle choice (2BC) paradigm. We analyzed cerebral cortices (CTX) and midbrains (MB) from male C57BL/6J mice subjected to a CIE, 2BC paradigm, which induces heavy drinking and represents one of the best available animal models for alcohol dependence and relapse drinking. CIE induced significant changes in protein levels in dependent mice compared with their non-dependent controls. Multiple protein isoforms showed region-specific differential regulation as a result of post-translational modifications. Our integrative analysis identified modules of co-expressed proteins that were highly correlated with CIE treatment. We found that modules most related to the effects of CIE treatment coordinate molecular imbalances in endocytic- and energy-related pathways, with specific proteins involved, such as dynamin-1. The qRT-PCR experiments validated both differential and co-expression analyses, and the correspondence among our data and previous genomic and proteomic studies in humans and rodents substantiates our findings. The changes identified above may play a key role in the escalation of ethanol consumption associated with dependence. Our approach to alcohol addiction will advance knowledge of brain remodeling mechanisms and adaptive changes in response to drug abuse, contribute to understanding of organizational principles of CTX and MB proteomes, and define potential new molecular targets for treating alcohol addiction. The integrative analysis employed here highlight the advantages of systems approaches in studying the neurobiology of alcohol addiction.

## Introduction

Prolonged alcohol exposure can result in a wide range of adaptive responses of neurons, changes in brain function, and significant brain damage [1], [2]. Changes in gene/protein expression patterns have been reported in a number of alcohol-related studies [3]. The majority of these studies has described the effects of alcohol on cerebral cortex (CTX), midbrain (MB), or their sub-regions from human, rat, and mouse brain, due to the particular susceptibility of these regions to the effects of long-term alcohol consumption and the related implications on cognitive, motor, executive functions [4]–[6], and on reward circuits associated with addiction [7]–[9]. One of the major limitations of previous gene expression studies is the fact that genes and their encoded proteins do not necessarily follow a parallel trend in expression levels. For example, alcohol administration can induce changes in protein translation without affecting mRNA levels [10]. Only about 75-85% of the transcript is translated into functional proteins, with sometimes even a lower correspondence [11]. In fact, changes in mRNA levels are not accurate predictors of altered protein expression [12]–[14].

Although previous proteomic studies in human alcoholics and animal models have identified candidate proteins, it is likely that alcohol dependence is the result of changes in co-regulation that might be invisible using single molecular-based analysis.

In addition, information on post-translational modifications (PTMs) of proteins can provide critical insight into how cellular processes are altered following alcohol abuse. Our group has recently shown the importance of utilizing novel systems-biology approaches to generate a comprehensive view of brain alterations in human alcoholics [15]. Here, we investigated the changes in protein expression levels, in CTX and MB from C57BL/6J mice subjected to a chronic intermittent ethanol (CIE), two bottle choice (2BC) paradigm, which induces heavy drinking and represents the best currently available animal model for alcohol dependence and relapse drinking. CIE is an established model involving repeated cycles of ethanol exposure that induces significant escalation of voluntary ethanol consumption [16]–[18].

Since the information captured by modern high-throughput techniques is far superior to any single molecular analysis, we chose a unique approach to data analysis that combines protein differential expression, protein coexpression networks, and gene annotations to advance an integrated molecular model of addiction.

## Materials and Methods

### Ethics Statement

All procedures were conducted in accordance with the guidelines established by the National Institutes of Health in the Guide for the Care and Use of Laboratory Animals and were approved by The Scripps Research Institute's Institutional Animal Care and Use Committee (Protocol Number: 11-0026).

### Chronic Intermittent Ethanol

**Figure 1:**
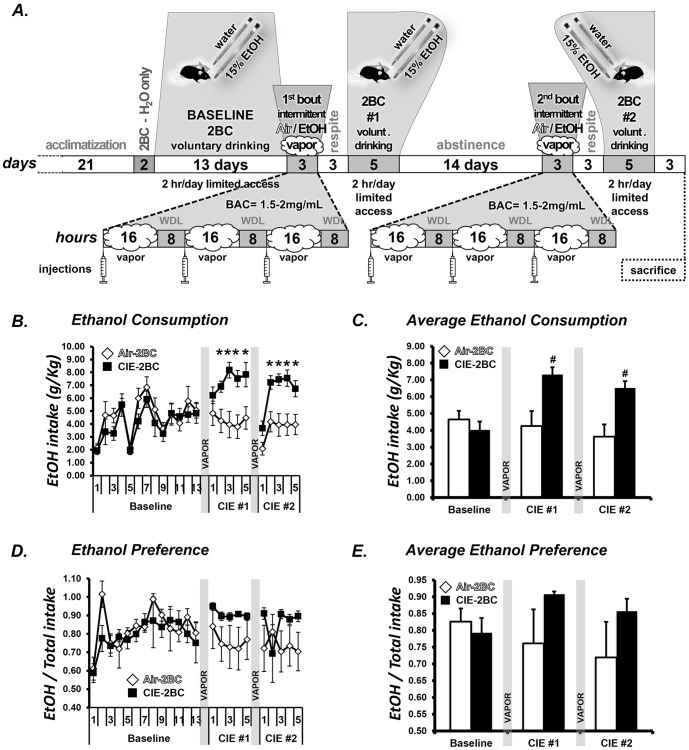
CIE effects on ethanol consumption and preference. **A**: General CIE protocol for 2BC drinking. Mice were made physically dependent on alcohol by intermittent EtOH vapor exposure (3X16h EtOH + 8 h Air). The EtOH consumption was measured during the 2 h limited access, 2BC procedure. The injections consisted of 68.1 g/kg pyrazole + saline or pyrazole + 1.5 g/kg 20% EtOH. Exposure to CIE paradigm increases EtOH drinking under 2BC conditions, compared to baseline responses. **B**: following the vapor/control chamber exposures (2BC#1 and 2BC#2) there were significant increases in EtOH consumption in CIE-2BC vapor-exposed mice relative to Air-2BC control mice on days 2-5. **C**: the average consumption shows a group effect after the two periods of drinking. **D**: alcohol preference was not significantly increased in CIE-2BC mice. **E**: average alcohol preference shows no significant group effect. *p<0.05; ^#^p<0.01 post-hoc analysis.

Previous studies have shown robust increases in drinking after repeated bouts of ethanol vapor exposure [16]. The actual paradigm that we used was based on the study published by Finn and colleagues [17]. Schematic overview of CIE paradigm is shown in Figure 1A.

Male C57BL/6J mice were acclimated to a reverse light cycle (6:00 am lights off, 6:00 pm lights on) for 21 days, food and water were available ad libitum throughout testing, and mice were group housed except during the alcohol drinking sessions. On days 1-13, three hours after the lights turn off (i.e., at 9:00 am), the mice were singly housed for two hours with access to two drinking tubes, one containing 15% ethanol and the other containing water. Ethanol and water consumption during these 2-hour periods were recorded. Following this baseline period, mice were divided, based on equal ethanol and water consumptions, into two balanced groups. The ethanol vapor group (CIE-2BC) received injections of a loading dose of ethanol (1.5 g/kg) and the alcohol dehydrogenase inhibitor, pyrazole (68.1 mg/kg in saline) before placement into the vapor chambers. The control group (Air-2BC) received pyrazole (68.1 mg/kg in saline) before placement into control chambers. Mice were placed in the chambers at 3:00 pm for 16 hours. At 7:00 am on the following day, mice were removed and tail blood sampled for blood alcohol determination. Vapor exposure was repeated on the following 2 days. Target BAC (blood alcohol concentration) was 1.5–2.0 mg/mL. Following the third day of exposure, mice were removed from the chambers at 7:00 am, tail blood samples collected, and mice were allowed 72 hours of undisturbed time. Both CIE-2BC and Air-2BC groups were then given 5 days of 2-hour access to bottles containing 15% ethanol and water to measure ethanol drinking and preference for the ethanol solution following ethanol vapor or control chamber exposure. Mice were then allowed a 2-week period of abstinence, and the vapor/control exposure and 5 days of two-bottle choice testing were repeated for a second period. A third group of mice (Naïve) had no access to EtOH bottles and were never exposed to vapor.

Brains were collected 72 hours after the last drinking session. Cerebral cortex (CTX) and midbrain (MB) were dissected from 7 CIE-2BC ethanol vapor-exposed (alcohol-dependent) mice, 7 Air-2BC air-exposed matched controls (which have also had access to alcohol), plus 7 ethanol-Naïve mice.

### Protein expression analysis

**Figure 2:**
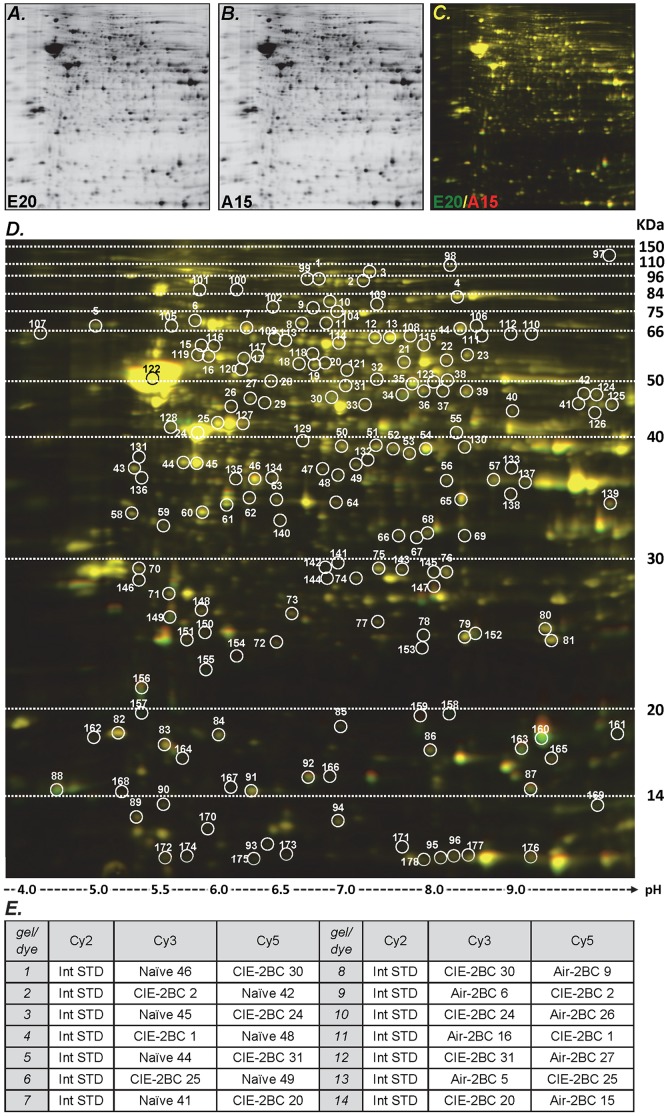
Example of 2D-DIGE gel. Gel images were scanned immediately following the SDS-PAGE. Each scan revealed one of the CyDye signals (Cy2, Cy3 and Cy5). Cy2 was used to normalize the signals from Cy3 and Cy5 channels. Overlay images were generated to compare different samples. The images shown were obtained from gel#14, CTX samples. **A**: proteome from sample E20, labeled with Cy3; **B**: proteome from sample A15, labeled with Cy5; **C**: overlay image generated from gel#14. In this case, a green spot represents an up-regulated protein in the CIE-2BC sample E20, compared to the Air-2BC sample A15. **D**: Overlay image and spot selection. Single and overlay images were generated, and a comparative analysis of all spots was performed using DeCyder “in-gel” or “cross-gel” analysis software. Spots 1-96 were selected based on 1.25-fold and p<0.1 for protein ratio cut-off, allowing for the appearance of the spots in 27 out of 28 gels (81 out of 84 total images). Spots 97-178 were added by lowering the cut-off to 1.15-fold for the significance threshold, without restricting the spots based on p-values; also, the stringency was lowered so that the spots appeared in at least 23 out of 28 gels (69 out of 84 appearances). **E**: Experimental design for 2D-DIGE gels. Two samples and the internal standard (Int STD) were loaded in each gel. Fourteen gels were run with CTX samples and an additional fourteen gels were run with MB samples. The randomized sample order and the dye-swap design avoided experimental biases.

Two-dimensional differential in-gel electrophoresis (2D-DIGE) was used to assess protein expression levels (Figure 2). Proteins were isolated from 21 cortices and 21 midbrains using the mirVana PARIS kit (Life Technologies) according to the manufacturer's instructions.

#### Preparation of samples

Protein samples were precipitated by methanol and then resuspended in 2-D cell lysis buffer (30 mM Tris-HCl, pH 8.8, containing 7 M urea, 2 M thiourea and 4% CHAPS). Protein concentration was measured using the Bio-Rad protein assay method (Bio-Rad, Hercules, CA).

#### CyDye labeling

Equal amounts of protein extract from randomly paired samples were labeled by CyDye DIGE fluors, size and charge matched respectively. The spectrally resolvable dyes enable simultaneous co-separation and analysis of samples on a single multiplexed (3 channels) gel. For each sample, 30 ug of protein was mixed with 1 µL of diluted CyDye and kept in the dark on ice for 30 min. The labeling reaction was stopped by adding 1 µL of 10 mM Lysine to each sample and incubating in the dark on ice for an additional 15 min. The labeled samples were then mixed together. 2X 2-D Sample buffer (8 M urea, 4% CHAPS, 20 mg/mL DTT, 2% pharmalytes and a trace amount of bromophenol blue), 100 µL destreak solution and Rehydration buffer (7 M urea, 2 M thiourea, 4% CHAPS, 20 mg/mL DTT, 1% pharmalytes and a trace amount of bromophenol blue) were added to the labeling mix to bring the total volume to 250 µL. The samples were mixed well and centrifuged before loading into the strip holder.

#### IEF and SDS-PAGE

An internal standard was created by mixing equal amounts of all 42 samples loaded in every gel using the Cy2 channel for alignment and cross-analysis of the gels. Up to three samples can be simultaneously separated on a single 2D gel, using isoelectric focusing (IEF) in the first dimension and SDS polyacrylamide gel electrophoresis (SDS-PAGE) in the second dimension.

After loading the labeled samples, IEF (pH3-10 Linear) was run following the protocol provided by GE Healthcare. Upon finishing the IEF, the IPG strips were incubated in freshly made equilibration buffer-1 (50 mM Tris-HCl, pH 8.8, containing 6 M urea, 30% glycerol, 2% SDS, trace amount of bromophenol blue and 10 mg/mL DTT) for 15 minutes with gentle shaking. Then the strips were rinsed in freshly made equilibration buffer-2 (50 mM Tris-HCl, pH 8.8, containing 6 M urea, 30% glycerol, 2% SDS, trace amount of bromophenol blue and 45 mg/mL iodoacetamide) for 10 minutes with gentle shaking. Next the IPG strips were rinsed in the SDS-gel running buffer before transferring onto 12% SDS-gels. Two samples and the internal standard (Int STD) were loaded in each gel. Seven gels included 7 CIE-2BC and 7 Air-2BC samples, while seven additional gels were used to test the same 7 CIE-2BC samples with 7 Naïve samples. A total of fourteen gels were used for CTX and an additional fourteen gels for MB. Sample order was randomized with a dye-swap design to avoid experimental biases (Figure 2E). The same pairing was used for both regions. The SDS-gels were run at 15°C until the dye front ran out of the gels. 2-D DIGE and Protein ID were performed by Applied Biomics, Inc (Hayward, CA).

#### Image scan

Gel images were scanned immediately following the SDS-PAGE using Typhoon TRIO (GE Healthcare, Little Chalfont, United Kingdom). Each scan revealed one of the CyDye signals (Cy2, Cy3 and Cy5). The scanned images were then analyzed by Image Quant software (v.6.0, GE Healthcare), including the single and overlay images (Figure 2A, B, and C).

#### Data analysis

Spot detection and matching were performed with a comparative cross analysis of all the gels using DeCyder software v.6.5 (GE Healthcare). Protein expression ratios between different samples or different groups of samples were calculated as originally described by Fodor [19]. Differential In-gel Analysis (DIA) module was used to co-detect and quantify the spots on a given gel, in terms of the ratios of the Cy3 and Cy5 sample volumes to the standard Cy2 volume. Biological Variation Analysis (BVA) module was used to match the spots and standardize the ratios across the gels, accounting for the observed differences in the Cy2 sample volumes [19]. Since the internal standard was identical on all gels, the software performed the matching only on the internal standard images labeled with Cy2, without introducing sample-to-sample differences into the matching [19]. The software selected a master gel as the gel with the most spots, and also provided as output a ratio of the normalized volumes, called standardized abundances. The statistical analyses in DeCyder are based on the standardized (divided by Cy2) protein log abundances, which are defined as the log10 of the standardized abundances. In theory, the standardized log abundances follow a normal distribution and are comparable across all spots and gels [19]. The ratios were calculated from the averages of the inverse logs, and the statistical significance was calculated with a t-test of the log std abundances and by checking the corresponding FDR. The standardized log abundances were also used for protein coexpression analysis.

96 spots were initially selected based on 1.25-fold and p-value <0.1 for protein ratio cut-off, allowing for the appearance of the spots in 27 out of 28 gels (81 out of 84 total images, best reproducibility). An additional 82 spots were added to the original selection by lowering the cut-off to 1.15-fold for the significance threshold without restricting the spots based on p-values; plus, the stringency was lowered so that the spots appeared in at least 23 out of 28 gels (69 out of 84 appearances) (Figure 2D).

### Protein identification by Mass Spectrometry

#### Spot picking and Trypsin digestion

The spots of interest were picked up by Ettan Spot Picker (GE Healthcare) based on the in-gel analysis and spot picking design by DeCyder software. The gel spots were washed a few times then digested in-gel with modified porcine trypsin protease (Promega, Fitchburg, WI). The digested tryptic peptides were desalted using a Zip-tip C18 (Millipore, Billerica, MA). Peptides were eluted from the Zip-tip with 0.5 µL of matrix solution (α-cyano-4-hydroxycinnamic acid, 5 mg/mL in 50% acetonitrile, 0.1% trifluoroacetic acid, 25 mM ammonium bicarbonate) and spotted on a MALDI plate.

#### Mass Spectrometry

MALDI-TOF MS and TOF/TOF tandem MS/MS were performed on AB SCIEX TOF/TOF™ 5800 System (AB SCIEX). MALDI-TOF mass spectra were acquired in reflectron positive ion mode, averaging 4000 laser shots per spectrum. TOF/TOF tandem MS fragmentation spectra were acquired for each sample, averaging 4000 laser shots per fragmentation spectrum on each of the 7-10 most abundant ions present in each sample (excluding trypsin autolytic peptides and other known background ions).

#### Database search

Both the resulting peptide mass and the associated fragmentation spectra were submitted to GPS Explorer workstation equipped with MASCOT search engine (Matrix Science, Boston, MA) to search the Swiss-Prot database. Searches were performed without constraining protein molecular weight or isoelectric point, with variable carbamidomethylation of cysteine and oxidation of methionine residues, and with one missed cleavage also allowed in the search parameters. Candidates with either protein score C.I.% or Ion C.I.% greater than 95 were considered significant. When multiple IDs were significant for a given spot, the selection was made by evaluating apparent molecular weight, isoelectric point, the location of the spot in the gel, and the presence of strips of multiple protein isoforms in the adjacent spots. 93 spots were identified with high confidence.

#### Data Availability

The MS data described in our manuscript have been deposited to the ProteomeXchange Consortium (http://proteomecentral.proteomexchange.org) via the PRIDE partner repository [20], with the dataset identifier PXD000349 and DOI 10.6019/PXD000349.

### Weighted Gene Correlation Network Analysis (WGCNA)

WGCNA is a tool for studying coexpression patterns (repeated trends of directional changes consistent across samples) in high throughput data [21], [22]. Similarity between expression patterns of two genes is assigned a weight (from 0 to 1). Correlation between genes tells how close their expression patterns are. Modules in coexpression networks are groups of interconnected genes showing over-represented patterns of coexpression and are detected by Linkage Hierarchical Clustering. Module Eigengenes (MEs) summarize and represent each module in one synthetic expression profile. We used MEs to treat modules as single units and relate them to external information used as trait (CIE- and Air-2BC phenotypes) via simple measures (correlation).

Standardized protein log abundances were subjected to coexpression analysis implemented in R environment using “WGCNA” package from Bioconductor. Expression data for 1,255 2D-DIGE gel spots (with 69 out of 84 gel appearances) were used.

The general framework of WGCNA has been described in detail previously [15], [21]. We ran separate analyses for proteins in each region. Briefly, Pearson's correlations were calculated for proteins and then a signed similarity parameter was derived, so expression profiles consist of the expression of protein spots across multiple 2D-DIGE samples. In the signed networks, the similarity between protein spots, reflects the sign of the correlation of their expression profiles. The signed similarities were then raised to power β (soft thresholding) to represent the connection strength with emphasis on high correlations at the expense of low correlations [22]. We chose the appropriate β softPower so that the resulting networks exhibited approximate scale-free topology. Next, all proteins were hierarchically clustered based on a dissimilarity measure of topological overlap which measures inter-connectedness for a pair of them [21]. The resulting dendrograms were used for module detection with the dynamic tree cut method (minimum module size, 10; cutting height, 0.995 and deepSplit = 2). Modules corresponding to the branches cut off of the protein tree were labeled in unique colors. Unassigned proteins were labeled in gray.

In our analysis, we related modules to CIE paradigm. As a trait, the “Escalation of Consumption” (EoC) trait was intended as increased ethanol consumption, with “0” for the Naïve group, “1” for Air-2BC, and “2” for CIE-2BC. We also used actual average ethanol drinking amounts for the last 5-days 2BC session.

### Real Time PCR analysis

Total RNA was isolated from the same 21 cortices and 21 midbrains used for the proteomic analysis, by using the mirVana PARIS kit (Life Technologies, Carlsbad, CA) according to the manufacturer's instructions. Yield and quality of the RNA was determined using a 2100 Bioanalyzer (Agilent, Palo Alto, CA). Single-stranded cDNA was synthesized from total RNA using the TaqMan™ High Capacity RNA-to-cDNA kits (Applied Biosystems, Foster City, CA). Following reverse transcription, quantitative RT-PCR (qRT-PCR) was performed in triplicate using TaqMan™ Gene Expression Assays (P/N: 4331182, Applied Biosystems) according to the manufacturer's instructions. All 7 samples for each experimental group were included in every reaction. The identification numbers for the single assays used are indicated in Table 1.

**Table 1:** Results of qRT-PCR analysis.

Comparison	mRNA	Encoded protein ID	Protein Spot #	2D-DIGE p-value	2D-DIGE ratio	TaqMan assay ID	RT-PCR p-value	RT-PCR FC	Ref. genes
Ctx, CIE-2BC/Naïve	*Dnm1*	DYN1	1	*3.50E-04*	1.24	Mm00802465_m1	*3.22E-03*	1.289	A, B
		DYN1	99	*2.40E-03*	1.59				
		DYN1	2	*4.60E-03*	-1.14				
Ctx, CIE-2BC/Naïve	*Fscn1*	FSCN1	121	*1.60E-03*	1.20	Mm00456046_m1	*3.91E-02*	1.186	A, B
Ctx, CIE-2BC/Naïve	*Flot1*	FLOT1	34	*1.20E-08*	1.38	Mm01275485_m1	*3.07E-05*	1.371	A, B
Ctx, Air-2BC/Naïve	*Snca*	SYUA	162	*3.60E-02*	1.25	Mm01188700_m1	*4.23E-02*	1.176	A, B, C
Ctx, Air-2BC/Naïve	*Dpysl2*	DPYL2	105	*1.70E-04*	1.22	Mm00515559_m1	*3.93E-01*	1.125	A, B, C
		DPYL2	109	*6.00E-04*	1.16				
		DPYL2	113	*1.90E-02*	1.17				
		DPYL2	114	*5.1E-03*	-1.25				

Confirmation of differential expression for selected mRNA with real-time PCR. Total RNA from cortex samples was used, and all 7 samples for each experimental group were included (number of datapoints per subgroup, n = 7). For DYN1 and DPYL2 values from different isoforms are listed. 2D-DIGE p-values are based on a t-test, and TaqMan assays p-values are based on an unpaired t-test, corrected for multiple testing. Data were normalized to the average of the endogenous control genes indicated (*Ref. genes*), based on qbasePLUS software's M scores. **A**, Gusb (Mm01197698_m1); **B**, Hprt1 (Mm00446968_m1); **C**, Tfrc (Mm00441941_m1). *ID*, Identification number; *FC*, fold change. P-values in italics, p<0.05.

RT-PCR was carried out in a ViiA™ 7 Real-Time PCR System (Applied Biosystems), data collected using ViiA™ 7 Software v. 1.2.2 (Applied Biosystems), and qRT-PCR results imported into qbasePLUS software v. 2.4 (Biogazelle, Zwijnaarde, Belgium) [23]. Data were normalized to the average of the best endogenous control genes based on their M scores calculated by the software (Table 1). Unpaired t-test with correction for multiple testing was used to assess statistical significance. Target correlation was calculated using Pearson correlation.

#### Functional annotations and bioinformatics tools

Differentially expressed and coexpressed proteins were submitted to the Database for Annotation Visualization and Integrated Discovery (DAVID; http://david.abcc.ncifcrf.gov/). Proteins were selected for functional annotation analysis based on differential expression with fold change ≥5% or ≤-5% p<0.2. Functional annotations of genes encoding differentially expressed or coexpressed proteins were also obtained by using Ingenuity Pathway Analysis (IPA) (Ingenuity Systems, www.ingenuity.com).

## Results

### CIE effects on ethanol consumption and preference

The groups were divided based on equal ethanol and water consumption; thus, there were no group differences across the baseline period (Figure 1B). Following the first vapor/control chamber exposure, there was a significant effect of group (F(1,14) = 9.6, p<0.01) (Figure 1C), with post-hoc analyses revealing increased ethanol consumption in ethanol vapor-exposed mice (CIE-2BC) relative to control mice (Air-2BC) on days 2-5 (p<0.05) (Figure 1B). Again, following the second chamber exposure there was a significant group effect (F(1,14) = 11.8, p<0.01), with post-hoc analyses revealing increased ethanol consumption in ethanol vapor-exposed mice relative to control mice on days 2-5 (p<0.05). While there was a trend for alcohol preference (ethanol intake divided by total fluid intake across the 2 hour periods) to be increased in ethanol vapor-exposed mice, this difference did not achieve statistical significance (Figure 1D, E). This was likely due, in part, to a ceiling effect in the vapor group. Blood alcohol concentrations (BACs) were 1.7+/-2.1 mg/mL during the first vapor exposure and 1.9+/-3.4 mg/mL during the second vapor period, in the range shown by Griffin and colleagues [18] to be critical for escalated ethanol drinking. Overall, this experiment was consistent with previous similar experiments, showing that repeated exposure to ethanol vapor is associated with subsequent increases in ethanol drinking under 2BC conditions [17].

### Protein differential expression

**Figure 3:**
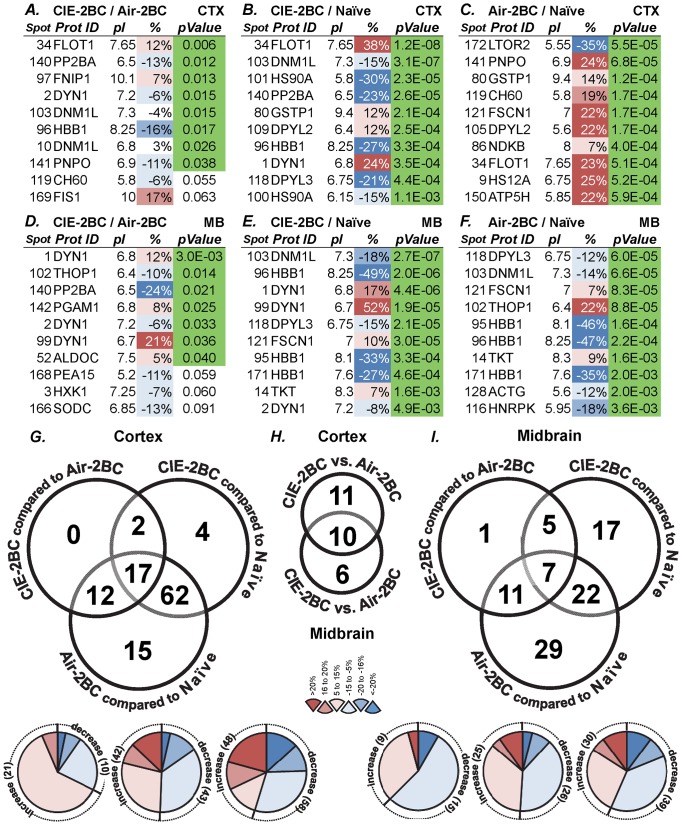
Protein expression changes induced by CIE paradigm. **A-F**, top 10 significant differentially expressed proteins for each comparison and brain region analyzed. **A-C**, cortex; **D-F**, midbrain. **A, D**: CIE-2BC vs. Air-2BC; **B, E**: CIE-2BC vs. Naïve; **C, F**: Air-2BC vs. Naïve. Different isoforms are listed for some proteins. *Prot ID*, protein ID; *pI*, isoelectric point. Refer to Methods for p-values and to Table S1 for the complete list of proteins. **G-H**, number of spots with differentially expressed proteins across specific group comparisons for CTX (**G**), MB (**I**), and between them (**H**). Number and direction of protein expression changes between CIE-2BC versus Air-2BC, CIE-2BC versus Naïve, and Air-2BC versus Naïve groups. The Venn diagrams indicate the number of shared and unique protein spots among comparisons. The pie charts show the amount of protein that increases and decreases within three ranges: 5-15%, 16-20%, >20% (absolute values); the numbers in brackets indicate the number of protein spots in that category. A total of 178 spots with stringency of 69/84 gels were considered. Only differences greater than 1.05 fold with p<0.2 are listed (Student's t-test). Most of the corresponding proteins have been identified.

Global proteomics was used to measure protein expression profiles in the CTX and MB of mice subjected to CIE-2BC or Air-2BC and alcohol-naïve mice (Figure 3). Following cross-analysis of 84 single channels from 28 gels, we selected 178 differentially expressed protein spots based on their statistical significance, fold change, and correlation across the comparisons (Table S1). We chose 95 spots of interest for mass spectrometry identification. After database searches, 92 protein IDs were identified with high confidence (C.I.% or Ion C.I.% >95) by analyzing spectra obtained from combined MALDI-TOF MS and TOF/TOF tandem MS/MS. One spot (#97) was identified with a lower confidence (Ion C.I.% = 88), and only 2 spots (#153 and #164) could not be identified.

We first identified the set of proteins that differed significantly between CIE-2BC and Air-2BC mice and found differential regulation in the alcohol-dependent versus non-dependent, low-drinking mice; for example, 31 proteins in the CTX and 24 proteins in the MB were differentially expressed in alcohol-dependent versus non-dependent mice (minimum fold change considered: ±5%, p<0.2). Half or more of these proteins were the same as those that differed from the Naïve group (Figure 3G, I). Then we identified proteins that were differentially expressed between CIE-2BC and alcohol-naïve animals and found 85 proteins in the CTX and 51 proteins in the MB that were differentially regulated. Finally, we identified different sets of proteins that are differentially expressed when non-dependent Air-2BC mice are compared with alcohol-naïve animals, indicating that proteins are rapidly changing in response to alcohol consumption in these mice; for example, 106 proteins in the CTX and 69 in the MB were differentially expressed in these groups (Figure 3G, I).

The most significant protein differences were found between the CIE-2BC versus the Naïve group (see Figure 3 for the top 10 differentially expressed proteins and Table S1 for the complete data of all 93 identified proteins). The differences were smaller when comparing CIE-2BC with their Air-2BC matched controls. Thirteen proteins were identified in more than one spot (e.g., dynamin-1, GAPDH, HBB1, STXB1, etc.) as multiple isoforms were resolved in the gels (Table S1).

### Dynamin-1 isoforms

**Figure 4:**
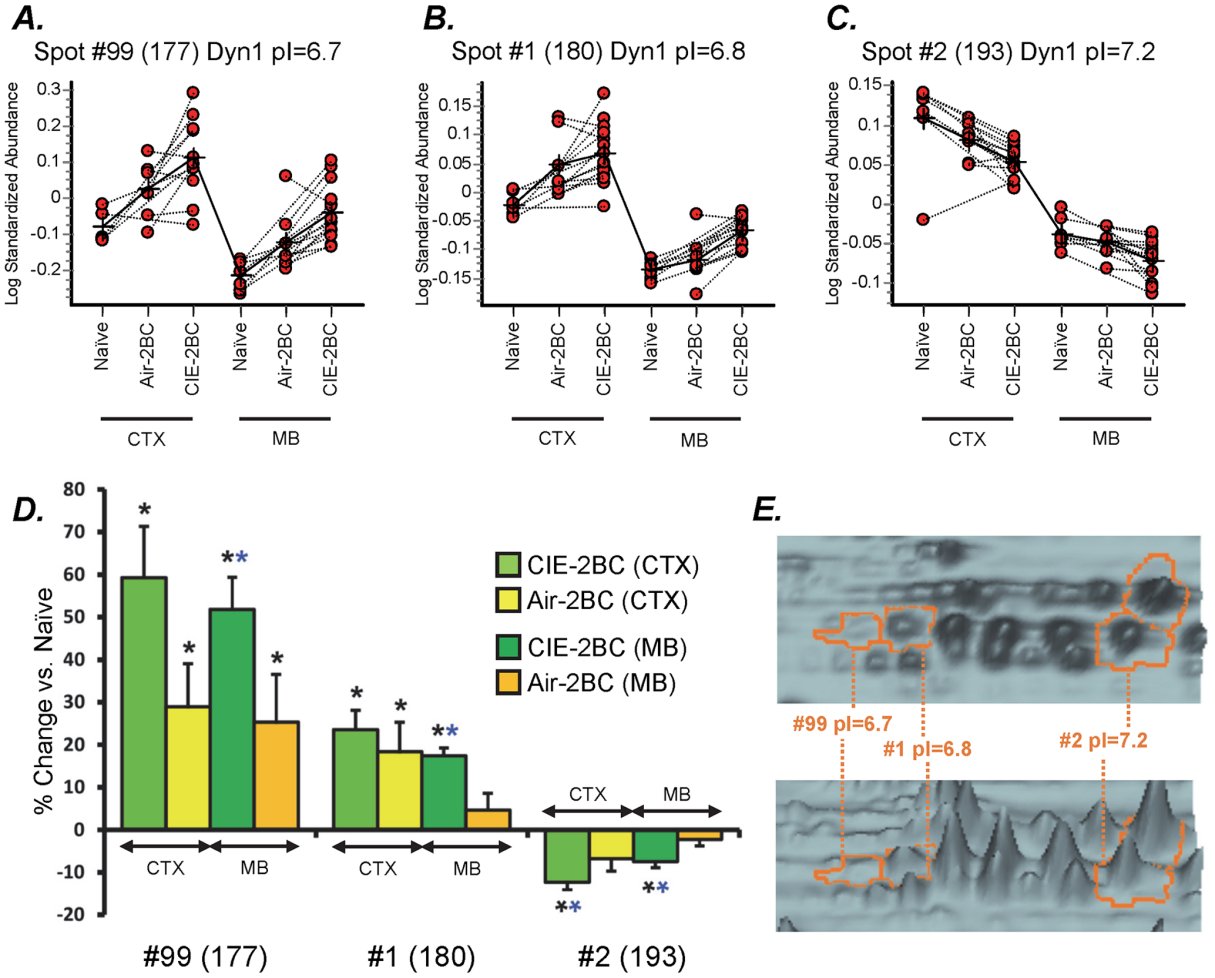
Differential expression of dynamin-1 isoforms in cortex and midbrain of CIE-2BC, Air-2BC, and Naïve mice. Isoforms with pI = 6.7 (**A**) and pI = 6.8 (**B**) show opposite regulation compared to isoform with pI = 7.2 (C). **A**, **B**, and **C** plots compare the relative abundance of the indicated isoform across different gels, compared to the corresponding internal standard. **D**: average normalized (divided by corresponding Naïve samples value) amounts of each isoform (derived from the average of at least 7 biological replicate samples per group) ± SEM. *p<0.05 vs Naïve; **p<0.05 vs Naïve and Air-2BC. **E**: Topographic displays of the gel area surrounding dynamin-1 isoforms on a representative sample from the CTX. Heights of the projections on the topographic display are representative of intensity of protein amounts. *Green*, CIE-2BC; *yellow*, Air-2BC.

Dynamin-1, -2, and -3 share 80% sequence homology and can all be found in mammalian brain tissue [24], [25]. Alternative splicing of the *Dnm1* gene produces six isoforms, while *Dnm2* and *Dnm3* both encode for two isoforms each. From our 2D-DIGE global proteomics analysis, we identified dynamin three times, in spots #1, #2, and #99 (apparent pIs 6.8, 7.2, and 6.7, respectively). Dynamin-1 expression was significantly altered in both CTX and MB following alcohol consumption. While two isoforms (pI = 6.7 and pI = 6.8 in our experimental conditions) showed an escalating up-regulation, another isoform (pI = 7.2) exhibited a gradual down-regulation during the transition from alcohol consumption to dependence in both CTX and MB (Figure 4, Table S1). The combined MS+MS/MS peptide sequence data were not able to clearly identify the exact dynamin isoforms present in the gel spots. Spot #99 contained a unique peptide (LNSPQGKHENR) from DYN1 isoform 1 (P39053-1), as well as two peptides present only in DYN2 and DYN3 sequences; spot #1 contained unique peptides for both DYN1 and DYN2 (not isoform-specific); spot #2 contained unique peptides for both DYN1 and DYN3 (not isoform-specific). Thus, even though the overall CI% scores and the relative abundance in brain tissue undoubtedly point to dynamin-1, there are probably traces of the two other isoforms in the gel spots which co-migrated at the same location.

### Over-represented functional categories

Top IPA functional categories for differentially expressed proteins between CIE-2BC and Air-2BC mice in CTX include the following: immunological, inflammatory (p: 5.8E-04 - 0.04), neurological and psychological diseases (p: 9.9E-04 - 0.048), with protein refolding (p = 1.6E-04), post-translational modifications (p: 1.6E-04 - 0.048), transport (p = 7.5E-09), endocytosis of synaptic vesicles (p = 1.5E-08), and neuronal cell death (p = 7E-04) (Table S2.1A). These functions along with energy production, glycolysis (p = 3E-04), hematological disease (p: 7.7E-05 - 0.03), and cancer (p: 8E-05 - 0.046) were found in MB (Table S2.1D). A comparison between regions shows greater adaptations in cellular assembly, function, and organization in CTX, with more differentially expressed proteins involved in related functional categories (Table S2.3). Similar IPA analyses were carried out for other group comparisons in CTX and MB (Table S2.1B, C, E, F).

Since several proteins were differentially expressed across group comparisons, we also used IPA to separately identify the following unique proteins that were differentially regulated in CIE-2BC vs. Air-2BC, but not in CIE-2BC vs. Naïve: ACTG, FIS1, FNIP1, HNRPK, HSP7C, PCBP2, SYUA in the CTX and DPYL2, GBLP, HNRPK, HXK1, PGAM1, PNPO, SODC in the MB. Interestingly, enriched biological functions included cell death and survival, neurological disease, mitochondrial dysfunction, and endocytosis signaling in the CTX (Table S2.1G), as well as hematological disease, glycolysis, and superoxide radical degradation in the MB (Table S2.1H). Similarly, immunological and inflammatory diseases, protein synthesis, glucocorticoid receptor signaling, and heme/tetrapyrrole synthesis related pathways were enriched when we analyzed the proteins regulated in CIE-2BC vs. Naïve, but not in the Air-2BC vs. Naïve (ODPX in the CTX and ACLY, ANXA5, FKBP4, G3P, HEM2, HS90A, PEA15, PP2BA, SEPT7 in the MB) (Table S2.1I, J).

Differentially expressed protein lists were fed into the DAVID web resource. For CIE-2BC vs. Air-2BC, among gene ontology categories and keywords, phosphoprotein (p = 5E-04, Bonferroni corrected, FDR = 0.7%), nucleotide binding (p = 6E-03), and acetylation (p = 0.01) were enriched in the CTX and phosphoprotein (p = 1.4E-05, Bonferroni corrected, FDR = 0.02%), acetylation (p = 0.019, Bonferroni corrected, FDR 24%), and glycolysis (p<0.05, Bonferroni corrected) were enriched in the MB (Table S2.2A, D). DAVID gene ontology categories for the differentially expressed proteins in the other group comparisons in CTX and MB are reported in Table S2.2B, C, E, and F.

### WGCNA analysis

**Figure 5:**
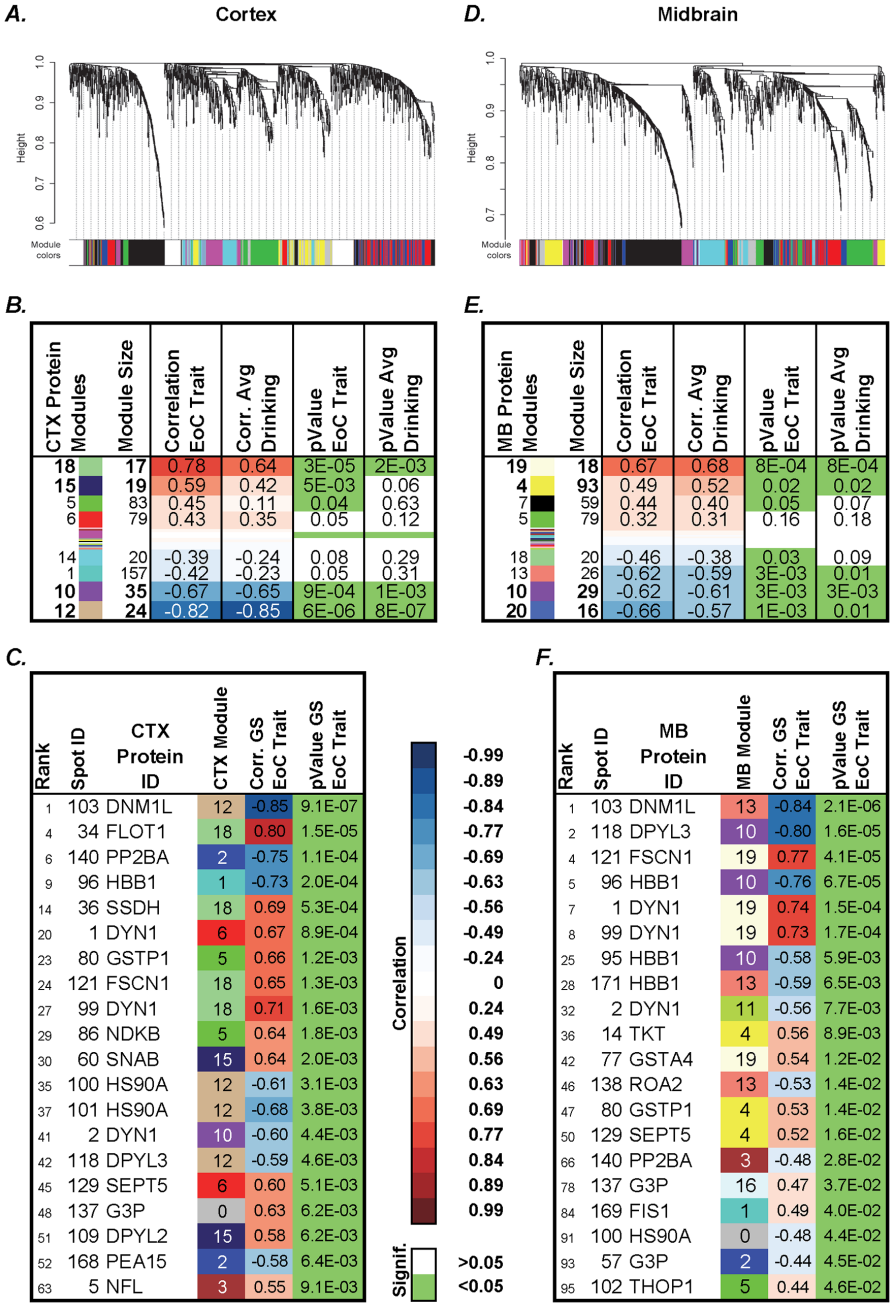
WGCNA analysis of protein expression in CTX and MB of animals subjected to CIE paradigm identified distinct modules of coexpressed proteins. **A** and **D** show dendrograms produced by average linkage hierarchical clustering. Horizontal color bars represent different coexpression modules. Bar sizes correspond to the number of proteins in each module. SoftPower β = 15 (**A**), 9 (**D**), minModuleSize = 10, cutHeight = 0.995, deepSplit = 2. Tables **B**, **E** show correlation (Corr.) between modules of coexpressed proteins and the EoC trait or the average 2BC ethanol consumption. Modules are named by a number and a color. Protein names are followed by corresponding gel spot number. Tables **C**, **F** show the relative contribution of proteins to CIE paradigm, in terms of correlation between the individual top 20 coexpressed proteins sorted by their gene significance (GS) for the EoC trait, with relative p-values and rank. Module number and color information are also included. In correlation columns, blue represents negative and red represents positive correlations, as reported on the legend. Green p-values are <0.05. Full lists are reported in Table S1.

Weighted gene coexpression network analysis (WGCNA) was performed on the standardized log abundances from 1,255 gel spots: Average linkage hierarchical clustering identified 19 distinct modules of coexpressed proteins in the CTX (Figure 5A) and 23 in the MB (Figure 5D). We related module eigengenes (MEs, see *Methods*) to CIE paradigm phenotypic data (“EoC” and drinking) used as trait through correlation analysis. In both regions, some modules are highly correlated with the 2BC EoC trait and the average drinking (for the last 5-days 2BC session).

Protein modules CTX18, CTX15, MB13, MB19, and MB4 are positively highly correlated (corr. = 0.49-0.78) with the EoC trait and the average ethanol consumption over the last 2BC session, while modules CTX10, CTX12, MB10, and MB20 were highly negatively correlated (corr.<-0.6) (Figure 5B, E). A list of the top 20 significantly coexpressed proteins is shown in Figure 5C, F.

### Validation by Real Time PCR analysis

To investigate translational regulation, we utilized qRT-PCR to measure expression levels of transcripts encoding those proteins differentially expressed due to CIE and/or 2BC drinking (Table 1). *Dnm1, Fscn1, Flot1*, and *Snca* mRNA levels were found significantly up-regulated in 2BC mice, consistent with their encoded protein levels, as shown by the global proteomic analysis. Interestingly, qRT-PCR expression levels for *Dnm1* correlated 0.81 with *Fscn1* levels (p = 3.3E-3) and 0.8 with *Flot1* (p = 3.3E-3). This is consistent with DYN1, FSCN1, and FLOT1 being in the same protein coexpression module (CTX18).

## Discussion

Alcohol abuse, tolerance, and physical dependence originate, at least in part, from multiple neuroadaptations in the brain, including widespread alterations in gene/protein expression patterns. Identification of these adaptations is complicated by the fact that alcohol is a complex trait disease that exerts its effects on multiple genes, although the effect of each gene may be quite small [26], [27]. This is why “omic” approaches have been extensively used to study altered patterns of gene/protein expression in brain following excessive alcohol exposure. Although the application of miRNomic, transcriptomic, and proteomic techniques in alcohol research has provided many useful datasets, our understanding of how individual expression changes work together to contribute to alcohol dependence is still quite limited. Indeed, these studies often lack the functional basis and contextual data necessary to formulate well-grounded hypotheses [15]. Systems biology and integrative analyses are thus crucial approaches to the future understanding of brain adaptations following drug abuse and dependence. Here, we present the results of global proteomics integrated with a system-wide approach involving coexpression analysis. Limitations in 2D-DIGE technology include reproducibility and relatively small dynamic range. Nevertheless, 2D-DIGE has the capability to provide profiling information on a large number of proteins and to efficiently separate protein isoforms for subsequent identification [28] and thus remains a vital method for detection of PTMed proteins.

The experimental design used in our study involved mice subjected to a CIE paradigm, which induced a significant escalation of 2BC ethanol voluntary consumption, associated with dependence [16], [18]. We observed smaller net effects, as expected, when comparing CIE-2BC with air-matched controls since both groups had access to alcohol in the 2BC model. We identified diverse sets of deregulated proteins in CTX and MB and showed that distinct sets of proteins were associated with alcohol consumption versus dependence (Figure 3, Table S1), suggesting critical neurobiological changes during the transition from alcohol consumption to dependence. Both 2BC-exposed groups consumed increasing levels of ethanol; however, CIE-2BC were subjected to higher brain alcohol concentrations compared with Air-2BC mice and experienced signs of alcohol dependence. The most significant differences were found between 2BC groups and Naïve animals (Table S1). This might suggest that the CIE exposure did not involve many additional proteins beyond what 2BC ethanol consumption produced, but rather exacerbated or reduced the effects on the same proteins. On the other hand, such result could also be interpreted as partially inherent to the technique used, with the same gel spots picked and analyzed across different conditions.

Many differentially regulated proteins from the present study have been described by previous genomic and proteomic studies in human alcoholic brain [15], [29]–[32] and rat [33]–[37] and mouse [38], [39] brain from animal models of alcohol abuse. Furthermore, some of the differentially expressed proteins reported here (i.e., DYN1, HSP7C, STXB1) were identified in our previous PPI study [40] as interacting partners of the BK channel, a well-established alcohol target which is important in behavioral and molecular tolerance to ethanol [41], [42].

The following proteins identified in our study were differentially expressed in human alcoholic frontal cortex: dynamin 1, syntaxin binding protein 1, heat shock 70–71 kDa proteins, glyceraldehyde 3-phosphate dehydrogenase, dihydropyrimidinase-related protein 2, guanine nucleotide-binding protein, neurofilament light polypeptide, septin, and creatine kinase [29]–[31]. Some of their encoding genes were also differentially expressed following excessive alcohol consumption [3], [15], [32], [43], [44]. We report changes in CTX and MB protein levels that suggest system-wide adaptive changes in energy metabolism, transport and endocytosis of synaptic vesicles, inflammatory response, PTMs and protein folding, nucleotide binding, blood oxygen-transport by hemoglobin, and cell death (Table S2) following alcohol dependence. Although similar functional categories were generally regulated in Air-2BC mice, we suggest that another regulation stage is linked to the onset of dependence; for example, flotilin-1 levels in Air-2BC are higher than in Naïve, but lower than in CIE-2BC mice. Several proteins identified from the studies above and our current work are involved in basal energy metabolism (e.g., creatine kinase, enolase, malate dehydrogenase, phosphoglycerate mutase 1, pyruvate kinase) [29]–[31]. Proteins involved in energy metabolism are regulated by many drugs of abuse as shown by neuroproteomic approaches [45]. Moreover, brain imaging studies in human alcoholics showed a marked reduction in whole brain glucose metabolism and modified brain resource allocation [46], [47]. Neurons adjust their intra- and extra-cellular environment in the presence of alcohol, and a disturbance of energy-generation pathways with related mitochondrial dysfunction could have profound consequences, including generation of reactive oxygen-nitrogen species [45]. Our results provide further support for the role of energy-related changes in two different alcoholic stages. Restoring impaired neuronal energy metabolism could represent a possible future direction for treatment of alcohol dependence.

Several differentially expressed proteins in 2BC-exposed groups were identified in more than one spot on the 2DIGE gels (i.e., dynamin-1, GAPDH). Some of them were separated in “strings” at the same molecular weight but with different isoelectric points (pI), although they have also been identified in isoforms of same pI but slightly different molecular weights (Table S1 and Figure 4). These multiple protein isoforms showed region-specific or isoform-specific differential regulation in different comparisons (Table S1) as a result of either alternate splicing or PTMs, contributing to the dynamic spectrum of the proteome. For example, we identified different isoforms of the 70 kDa heat shock protein in agreement with previous studies [30], [31]. Alcohol-stimulated induction of heat shock proteins (HSPs) may be part of a protective response against oxidative stress [48]. HSPs are also regulated by several other drugs of abuse at both the mRNA and protein levels [45]. Alcohol can activate the heat shock pathway through the up-regulation of HSF1-dependent genes, Hsp70 and Hsp90, in cultured neurons and in mouse cerebral cortex [49]. Our data suggest that isoforms of HSP60 and HSP70 are increased, while isoforms of HSP90 are decreased, in the cortex of CIE-2BC mice compared with the Naïve group (Table S1). Ethanol and heat shock response can also increase gene expression of *Syt1*, *Snap25* and *Vamp2*, members of the SNARE complex that is in turn associated with the BK channel and dynamin-1 [40]. These changes could also represent a counter-regulatory action against the potentially toxic cellular effects of alcohol, rather than a general stress-like situation following its abuse [45].

We also identified three different isoforms of dynamin-1, a protein involved in vesicular trafficking processes and receptor-mediated endocytosis [24], [25]. These isoforms showed an escalating up-regulation or a gradual down-regulation during the transition from alcohol consumption to dependence (Figure 4, Table S1). We also show evidence of significant up-regulation of *Dnm1* gene expression in the CTX by RT-PCR (Table 1). Dynamin-1 was previously shown to be up-regulated in the dorsolateral prefrontal cortex of cirrhotic and non-comormid alcoholics [50]. However, multiple isoforms of dynamin-1 have been reported as down-regulated in the superior frontal gyrus (SFG) and occipital cortex (OC) of chronic human alcoholics [30] but not in the SFG of cirrhotic alcoholics [31]. These differences might be due to the different sets of proteins isolated in our protein extract of whole mouse brain tissue versus the simple fraction of synaptosomes from human alcoholics. Second, in alcoholics, the brain has adapted to high concentrations of alcohol over many years, and a general miRNA up-regulation [51] is likely to cause decreased protein expression [32]. Finally, there is considerable variability in human brain compared to the mouse model, where the early stages of an induced dependence drive the mice to high alcohol consumption. Dynamin-1 (among other proteins involved in vesicle budding, docking, and fusion in cellular vesicular transport) has been linked to different addictive disorders in several proteomics studies [52]. Although vesicle transport/trafficking is ubiquitous, this function is crucial for fine-tuning neurotransmission. Aside from being the major mechanism for synaptic vesicle recycling in the presynaptic compartment and trafficking of postsynaptic receptors, it is also fundamental in neuronal cellular physiology. Changes in dynamin protein expression may not only affect the efficiency of synaptic transmission but may also influence neuronal size and architecture. Indeed, repeated exposure to addictive substances usually leads to persistent changes in synaptic plasticity in certain neurons [52], [53].

**Figure 6:**
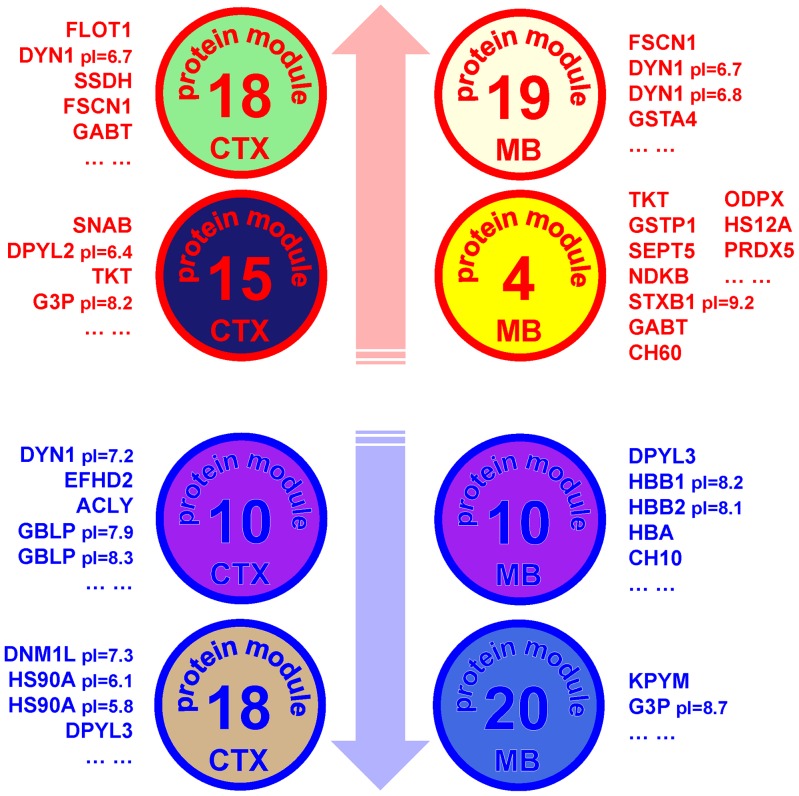
Proteins identified from the top two positively and negatively correlated modules. Proteins and module colors are derived from WGCNA and correlation analysis in cortex and midbrain. These proteins respond to 2BC drinking and/or CIE with opposite directional changes in their expression levels. Proteins in modules positively correlated to the EoC trait show a parallel concomitant gradual up-regulation in their expression levels, while those in negatively correlated modules are down-regulated when mice consume more ethanol. *Circles*, protein modules; *blue and red*, negative and positive correlation with the EoC trait.

We used a sophisticated systems approach to data analysis and applied WGCNA [22] to identify groups of proteins showing over-represented patterns of coexpression. Some of these distinct modules of coexpressed proteins (Figure 5B, E) showed a high correlation with the EoC trait and alcohol drinking in CTX and MB. Modules showing the highest positive correlation to the EoC trait include proteins involved in energy metabolism and endocytosis signaling (CTX15, CTX18, MB4, MB19) (Figure 6). The significance of our WGCNA analysis is verified by the RT-PCR experiments, where we tested the expression levels of three genes (*Dnm1*, *Fscn1*, *Flot1*) encoding coexpressed proteins from the same module and obtained high Pearson correlations among their expression patterns across the samples. Furthermore, differentially expressed proteins are often over-represented in top correlated protein modules in both CTX and MB (p<0.05 hyper geometric test) (Table S3), emphasizing the complementarity of differential expression and coexpression as analytical tools for detecting coordinated molecular changes.

Our analysis did not fully cover the temporal evolution of gene network responses, due to the limited number of experimental groups utilized. Furthermore, the design of the study did not allow for distinguishing expression changes that are related to forced exposure to ethanol vapor versus voluntary consumption of alcohol. Future studies should incorporate an experimental group of mice exposed only to ethanol vapor and brain samples collected at different time points.

In summary, our large-scale analysis of different brain regions from mice subjected to a CIE paradigm reveals the following: 1) significant changes in protein expression, 2) coordinated regulation of proteins, and 3) expression differences during the transition from ethanol consumption to dependence. We introduce an integrative systems approach that will advance knowledge of brain remodeling mechanisms and adaptive changes in response to drug abuse. To date this expansive approach is unsurpassed in alcohol studies and opens a new avenue in addiction research. Importantly, our findings identify key molecules that appear to regulate neuronal adaptations associated with alcohol dependence, thus highlighting potential molecular targets for treating alcohol abuse.

## Supporting Information

Table S1 **Mass spectrometry identification and semi-quantitative ratios for 93 differentially expressed protein spots.** The spots of interest were detected based on the in-gel analysis and spot picking design by DeCyder software. MALDI-TOF MS and TOF/TOF tandem MS/MS were performed and resulting peptide mass and the associated fragmentation spectra were submitted to MASCOT search engine. Candidates with either protein score C.I. % or Ion C.I. % greater than 95 were considered significant. The best matches were selected based on C.I.% and pI/MW location of the spot in the gel. The dataset provides an overview of the semiquantitative ratios, p-values, and FDR values for each group comparison of interest, as calculated by DeCyder software (see Methods). Relative contribution of each protein to the EoC trait is also shown, as calculated by WGCNA analysis of protein expression in cortex and midbrain of mice subjected to CIE paradigm. WGCNA-related columns show correlation between individual proteins and the EoC trait, with relative p-values and rank. Module information are also included: Number, color, and frequency (size). In correlation columns, blue represents negative and red represents positive correlations. Green p-values are <0.05.
(XLS)

Table S2 **Tab 1. IPA analysis of differentially expressed proteins.** IPA summary for differentially expressed proteins across multiple comparisons. A-C, G, I, cortex; D-F, H, J, midbrain. **A**, **E**: CIE-2BC vs. Air-2BC; **B**, **E**: CIE-2BC vs. Naïve; **C**, **F**: Air-2BC vs. Naïve. **G**, **H**: CIE-2BC / Air-2BC minus CIE-2BC / Naïve; **I**, **J**: CIE-2BC / Naïve minus Air-2BC / Naïve. Proteins used for the analysis were selected from cortex and midbrain based on fold change ≥5% or ≤-5% and p<0.2, excluding duplicates due to isoforms. The following data are reported: top networks, top biological functions, top canonical pathways, and biological functions, listed with their respective scores, p-values, and number of molecules involved. For the top canonical pathways, ratios provided represent the number of genes from the dataset that map to the pathway divided by the number of all known genes ascribed to the pathway. **Tab 2.** DAVID functional analysis of differentially expressed proteins. Top functional annotations for differentially expressed proteins across multiple comparisons. **A**-**C**, cortex; **D**-**F**, midbrain. **A**, **D**: CIE-2BC vs. Air-2BC; **B**, **E**: CIE-2BC vs. Naïve; **C**, **F**: Air-2BC vs. Naïve. Proteins used for the analysis were selected from cortex and midbrain based on fold change ≥5% or ≤-5% and p<0.2, excluding duplicates due to isoforms.
(XLS)

Table S3 **Over representation of DE proteins in coexpression modules.** Protein coexpression modules in cortex (**A**) and midbrain (**B**) sorted by correlation and related with protein differential expression. The number of differentially expressed proteins present in the module was tested for over representation with a hyper geometric test (protein background size  =  1255, DE proteins checked in background =  top 100 for CTX or top 50 for MB). Differential expression data from all comparisons were tested: CIE-2BC vs. Air-2BC; CIE-2BC vs. Naïve; Air-2BC vs. Naïve. Related IPA functional annotations are indicated for each module. In correlation columns, blue represents negative and red represents positive correlations. In p-value columns, light green indicates p<0.05, and dark green p<0.01. Module size, number of proteins in the module; *Corr*., correlation; *DE Proteins*, number of differentially expressed proteins (in the relative comparison) in the module; *Hyperg. p-Value*, p-value of the hyper geometric test; *IDed Proteins*, number of proteins identified within the module.
(XLS)
